# Detritus Quality and Locality Determines Survival and Mass, but Not Export, of Wood Frogs at Metamorphosis

**DOI:** 10.1371/journal.pone.0166296

**Published:** 2016-11-08

**Authors:** Joseph R. Milanovich, Kyle Barrett, John A. Crawford

**Affiliations:** 1 Department of Biology, Loyola University Chicago, Chicago, Illinois, United States of America; 2 Department of Forestry and Environmental Conservation, Clemson University, Clemson, South Carolina, United States of America; 3 Department of Biological Sciences, Lindenwood University, St. Charles, Missouri, United States of America; Consejo Superior de Investigaciones Cientificas, SPAIN

## Abstract

Single-site experiments have demonstrated detritus quality in wetlands can have strongly negative, neutral, and even positive influences on wildlife. However, an examination of the influence of detritus quality across several regions is lacking and can provide information on whether impacts from variation in detritus quality are consistent across species with wide ranges. To address this gap in regional studies we examined effects of emergent and allochthonous detritus of different nutrient qualities on amphibians and assessed a mechanism that may contribute to potential impacts. We used aquatic mesocosms to raise wood frogs (*Rana sylvatica*) from two regions of the United States with whole plants from purple loosestrife (*Lythrum salicaria*), leaf litter from native hardwood trees, and a mixture of both. We examined several metrics of amphibian fitness and life history, including survival, number of days to metamorphosis, and size at metamorphosis. Further, we quantified whether the effects of detritus type could translate to variation in anuran biomass or standing stock of nitrogen or phosphorus export. Our results show detritus with high nutrient quality (purple loosestrife) negatively influenced survival of wood frogs, but increased size of metamorphic individuals in two different regions of the United States. Despite the decrease in survival, the increase in size of post-metamorphic anurans raised with high quality detritus resulted in anuran biomass and standing stock of N and P export being similar across treatments at both locations. These results further demonstrate the role of plant quality in shaping wetland ecosystem dynamics, and represent the first demonstration that effects are consistent within species across ecoregional boundaries.

## Introduction

Availability and quality of resources early in life can shape fitness [1,2] and a variety of life history traits of organisms, such as morphology [3,4] and size [5,6,7]. In particular, bottom-up effects can have measurable impacts on organisms with complex life histories, such as those that undergo metamorphosis. Factors such as food limitation, resource quality, density, competition and physiology can all play roles in an individual’s development [8]. As anthropogenic changes continue to increase [9,10,11], replacement, gains or losses of producers can have considerable effects on consumer composition and ecosystem processes [12,13]. Thus, as global change alters resources in ecosystems it is important to understand the consequences and assess the extent to which effects vary across broader spatial scales.

Anthropogenic changes to plant composition are predicted to have measurable effects on wetland ecosystem structure and function [14,15]. Primary productivity in temperate wetlands is generally low [16] and wetlands are often characterized as nutrient limited [17] due to their reliance upon allochthonous input to provide much of the available resources (e.g. senescent leaves). Changes in the quality of resources available to wetlands are driven by plant traits along two different axes. Plant quality (and ultimately, effects on consumers) varies in both nutrient composition and the concentration of secondary compounds [18,19]. As global change continues, the composition of plant species is predicted to change [20,21]. If compositional change occurs it could drive changes in plant quality by either increasing populations of lower quality invasive species or by replacement of current native species by adjacent native species better adapted to the changing climate. Larval anurans are likely to be strongly affected by changes to resource quality in wetlands, as these organisms feed across trophic levels and are heavily reliant on allochthonous productivity in wetland ecosystems [22]. Recent studies have shown plant traits such as carbon-to-nitrogen ratios and variation in leaf litter species can impact development, morphology and fitness of larval anurans [3,4,6,23,24,25]. Martin et al. [25] suggest lower leaf litter C:N contributes to faster decomposition and supports higher quality biofilms, which result in higher tadpole performance. Other plant traits, such as secondary plant compounds effect larval anuran development and survival directly via toxicity [2,26] or indirectly by reducing food availability (e.g., biofilms and zooplankton, [27,28]).

Taxa with biphasic lifecycles, such as amphibians, directly link changes in wetland resource quality (i.e. variation in plant quality) to terrestrial systems. Larval anurans raised in environments with varying resource quality (e.g., variation in nutrient content and secondary compound concentrations) provide different quantities of subsidies (biomass and nutrient) to the surrounding upland environment via metamorphosis [29,30,31]. For example, Earl et al. [32] found that survival, size and biomass export of gray treefrogs (*Hyla versicolor*) was strongly affected by leaf litter species and associated plant traits. Thus, variation of detritus quality driven by global climate change may alter the amount and direction of flow in reciprocal subsidies between ecosystems.

Our objectives of this study were to examine the influence of high and low nutrient quality detritus on the fitness and life history (survival, development and size), biomass and standing stock of N and P export of wood frogs (*Rana sylvatica*). In our study, high quality nutrient detritus was represented by alien purple loosestrife (*Lythrum salicaria*) and low quality nutrient detritus by a mixture of native hardwood leaf litter. To increase validity of potential effects across a single species, we conducted the experiments in two regions where wood frog populations are at the periphery of their distributional range. At each location we hypothesized larval anurans raised with high nutrient quality detritus would have increased survival, size and biomass export, and decreased developmental time relative to wood frogs raised in low nutrient quality plant treatments.

## Methods

### Study species

Wood frogs occur from the southern Appalachian Mountains of Georgia, north above the Arctic Circle, and west to Alaska. The western border in the contiguous United States ranges from Missouri to North Dakota and the southern border extends from southern Illinois to northern Georgia, U.S.A. In Missouri and South Carolina breeding typically occurs between January and March in fish-free, ephemeral woodland pools or ponds [33]. Wood frogs complete metamorphosis between days 65 to 130; prior to metamorphosis larvae feed primarily on detritus, plant/algal material and some zooplankton [22,33,34]. Following metamorphosis, adults are typically found in forested landscapes [33].

### Experimental unit design

We established completely randomized tank experiments at Lindenwood University’s Daniel Boone Field Station (St. Charles County, Missouri, U.S.A.; located at the southwest periphery of wood frogs range; 38.7865°N, 90.5039°W) and Clemson University’s Environmental Toxicology facility (Anderson County, South Carolina, U.S.A; located at the southeast periphery of wood frogs range; 34.6805°N, 82.8392°W) to examine the response of larval anuran developmental time, size at metamorphosis, and survivorship when raised with two different detritus qualities. At each site, we replicated three detritus treatments with wood frogs six times for a total of 18 experimental units. In December 2013 we stocked 133 L tanks (Beckett Corporation, Irving, TX; ~30 cm water depth and 99 cm diameter) with approximately 120 L of tap water. Next, we used t-tests to examine whether detritus nutrient content (N, P or C:N ratio; homogenised mixture of native leaf litter [n = 5] or purple loosestrife [n = 5]) differed within and between each site (Clemson or Lindenwood). The %N, %P or C:N of mixed hardwood native hardwood leaf litter (see below for details) did not significantly differ between Clemson and Lindenwood (*P*≥0.05 for all t-test; see below), but %N, %P and C:N of mixed hardwood native leaf litter from each site and alien purple loosestrife significantly differed (*P*≤0.05 for all t-test; see below); therefore, we created three treatments representing 150 g of high nutrient litter quality (whole purple loosestrife plant detritus; herein high), 150 g of low nutrient quality detritus (mixture of hardwood leaf litter from each site; herein low) and a 150 g mixture of both purple loosestrife (75 g) and hardwood leaf litter (75 g), herein mixed. This provided a detritus density similar to previous experiments [4,18], but a lower density than other mesocosm studies [35,36,37]. Tanks were then covered with 60% shade black mesh cloth to simulate moderate canopy cover and prevent colonization of invertebrates. After 24 hrs, 1 L of natural wetland water from wetlands (where amphibian egg masses were subsequently collected) was added to inoculate the tanks with microbes and zooplankton from respective sites. Native hardwood litter was collected immediately after senescence during autumn 2013 from the riparian area of wetland sites where anuran egg masses were subsequently collected (St. Charles County, Missouri or Pickens County, South Carolina) and was allowed to dry indoors until placement in tanks. To stock mesocosms (see below; herein tank), we used mixed hardwood native leaf litter consisting of scarlet oak (*Quercus coccinea*), white oak (*Quercus alba*) and American sweetgum (*Liquidambar styraciflua*) within Clemson tanks and black oak (*Quercus velutina*), post oak (*Quercus stellata*), and white ash (*Fraxinus americana*) within Lindenwood tanks.

Whole purple loosestrife plants were collected immediately after senescence during autumn 2013 from a single wetland location (to standardize the influence of plant quality) in Porter County, Indiana, U.S.A. Plants were bagged in large plastic garbage bags and were allowed to dry indoors at Loyola University Chicago until placement in tanks. Although using purple loosestrife from a single location eliminated variability in plant quality, we do not know if the litter quality of this population was typical. For example, purple loosestrife at this single location may have higher secondary compounds compared to other locations.

In spring 2014, approximately 10 days prior to introduction of anuran larvae, we again inoculated tanks with 1 L of natural wetland water from sites where anuran egg mass were collected and 5 g of Purina Rabbit Chow to stimulate production of phytoplankton and zooplankton [18]. Due to the differences in phenology between the two sites, three to four egg masses of wood frogs were collected from a single wetland at each location during the breeding season (February—March 2014 for Clemson and March—April 2014 for Lindenwood). Masses were retained in holding tanks (as described above for low nutrient quality detritus treatment) at the location where the experimental tanks were placed (see above) until animals hatched and reached Gosner stage 21 (free swimming with external gills still present; [38]). When animals reached the desired developmental stage, 20 larvae were randomly allocated from the holding tanks to each of the three vegetation treatments described above (n = 360 larvae per site). In each tank approximately 0.5 X 0.1 m of foam was placed inside to provide habitat for metamorphic animals [39].

### Anuran fitness

Following placement of anuran larvae in tanks, larvae were allowed to develop until metamorphosis (Gosner stage 44; only tail bud remaining) without being handled; therefore, larvae were not monitored following placement until metamorphosis began. Once larvae approached stages near metamorphosis, tanks were checked daily to collect animals immediately at this developmental stage. Upon collection after metamorphosis, we initiated a humane endpoint and animals were sacrificed by being placed in a solution containing 1 L of de-chlorinated water and 2 grams of maximum strength Orajel® (Active ingredient: 20% benzocaine; following methods of [40]). We did not account for acidity and poor solubility of benzocaine in water during euthanasia, as this was not a requirement of ethics committees at Lindenwood University or Clemson University. We recorded the total number of days in the mesocosm (e.g., time to metamorphosis) and individuals were weighed (to the nearest g) and measured (snout-to-vent length; SVL; to the nearest mm). In the event that a metamorphic animal was found dead (16 individuals [4.4% of all individuals] at Lindenwood and 21 individuals [5.8% of all individuals] at Clemson), it was assumed the animal completed metamorphosis the prior day and died as a result of drowning and was not counted as a death in survival estimates. Frequency of monitoring did not vary from once per day following estimated drownings. At the time the animal was found dead the animals were feeding independently within each tank. Survival was calculated as the proportion of surviving individuals that completed metamorphosis. Metamorphic individuals were weighed and SVL was measured. The experiment ended at both sites when 14 days passed without a metamorphic animal found, and at this time all mesocosms were drained and searched for larvae that did not metamporphose (all surviving individuals metamorphosed). The tanks were not examined for health of animals following their placement into the tanks prior to the experimental endpoints (see above) to minimize disturbance.

To provide a measure of nutrient biomass export from each tank, we examined the whole-body stoichiometry of the first two anurans and last two anurans within each tank that reached metamorphosis (Gosner stage 44) and various plant samples at Clemson and Lindenwood. Each individual anuran was processed as above, dissected to remove the stomach and intestinal tract, and frozen. Five samples of native hardwood from Clemson and Lindenwood, and five samples of purple loosestrife stems, seed pods and leaves (homogenized) were collected prior to placement into tanks to provide a measure of the nutrient composition of plant material within each tank. Carcasses and litter were oven dried at 60°C for seven days to a constant weight, homogenized, weighed into tin capsules and analyzed for whole-carcass/homogenised plant %C and %N using Micro-Dumas Combustion using a Carlo Erba 2NA 1500 CHN analyser (Carlo Erba, Milan, Italy). Carcass and litter %P was measured by weighing samples into acid-washed and pre-ashed ceramic crucibles, ashed at 500°C, acid digested and analysed spectrophotometrically (ascorbic acid method). Ground pine needles (US National Institute of Standards and Technology, 1575a) and poplar leaves (Analytical Chemistry Laboratory, University of Georgia) were used as external standards for P analyses. Carbon, N and P analyses were conducted by the Analytical Chemistry Laboratory at the University of Georgia, U.S.A. Lastly, metamorphic anuran biomass was quantified for each tank by taking the product of mean dry mass and survival within each tank. Mean dry mass was used as the dry mass of each metamorphic individual was not determined (see above); therefore, a mean of the individuals in the tank used in stoichiometric analysis was used to calculate standing stock of biomass, N and P to eliminate the influence of wet mass measurements. Standing stock of N and P within metamorphic anurans was calculated by the product of biomass and mean whole-body %N or %P.

This project, including euthanasia using Orajel®, conformed to the legal requirements for the use of vertebrates in research [41] and was approved by Clemson’s Institutional Animal Use and Care Committee (AUP 2013–066). Furthermore, Lindenwood University animal care methods followed protocols from Guidelines for Use of Live Amphibians and Reptiles in Field Research compiled by the American Society of Ichthyologists and Herpetologists, The Herpetologist League, and the Society for the Study of Amphibians and Reptiles (document can be accessed at: http://www.aaalac.org/accreditation/Guidelines_for_Use_of_Live_Amphibians_and_Reptiles.pdf). The collection of purple loosestrife was conducted under Indiana Division of Nature Preserves permit NP13-49 and Illinois Department of Natural Resources permit NH14.5757. Egg mass collection permits were obtained from South Carolina Department of Natural Resources and the Missouri Department of Conservation (Permit # 15978).

### Statistical analyses

We used general linear models to determine whether survival was predicted by site (Clemson and Lindenwood; fixed effect), treatment (high or low nutrient quality detritus; fixed effect) or the interaction of site and treatment. Normal probability plots of residuals for survival suggested normality. An analysis of covariance (ANCOVA) model was used to determine whether time to metamorphosis or size (wet mass) was significantly different across sites or treatment (fixed effects) when accounting for survival within each tank (covariate). Levene’s test was used to check for homogeneity of variance among groups before running the ANCOVAs (and in all cases, we met the requirements of the parametric test). Two-way ANOVAs were used to examine whether whole-body post-metamorphic anuran stoichiometry (N, P, C:N and N:P ratio), biomass, or standing stock of anuran N and P differed across sites (fixed effect) or treatments (fixed effect), or if there was an interaction between site and treatment. Whole-body post-metamorphic anuran N and C:N stoichiometry data were not normally distributed (even following transformation); however, evidence suggests ANOVAs are robust against non-normal data [42]. To further support two-way ANOVAs with non-normal data we used Mann-Whitney U tests to examine whole-body %N and C:N between sites within each litter treatment. All data were normally distributed unless otherwise noted and all statistical analyses were performed using STATISTICA 12.0 (Statsoft, Inc., Tulsa, OK).

## Results

### Anuran fitness

**Fig 1:**
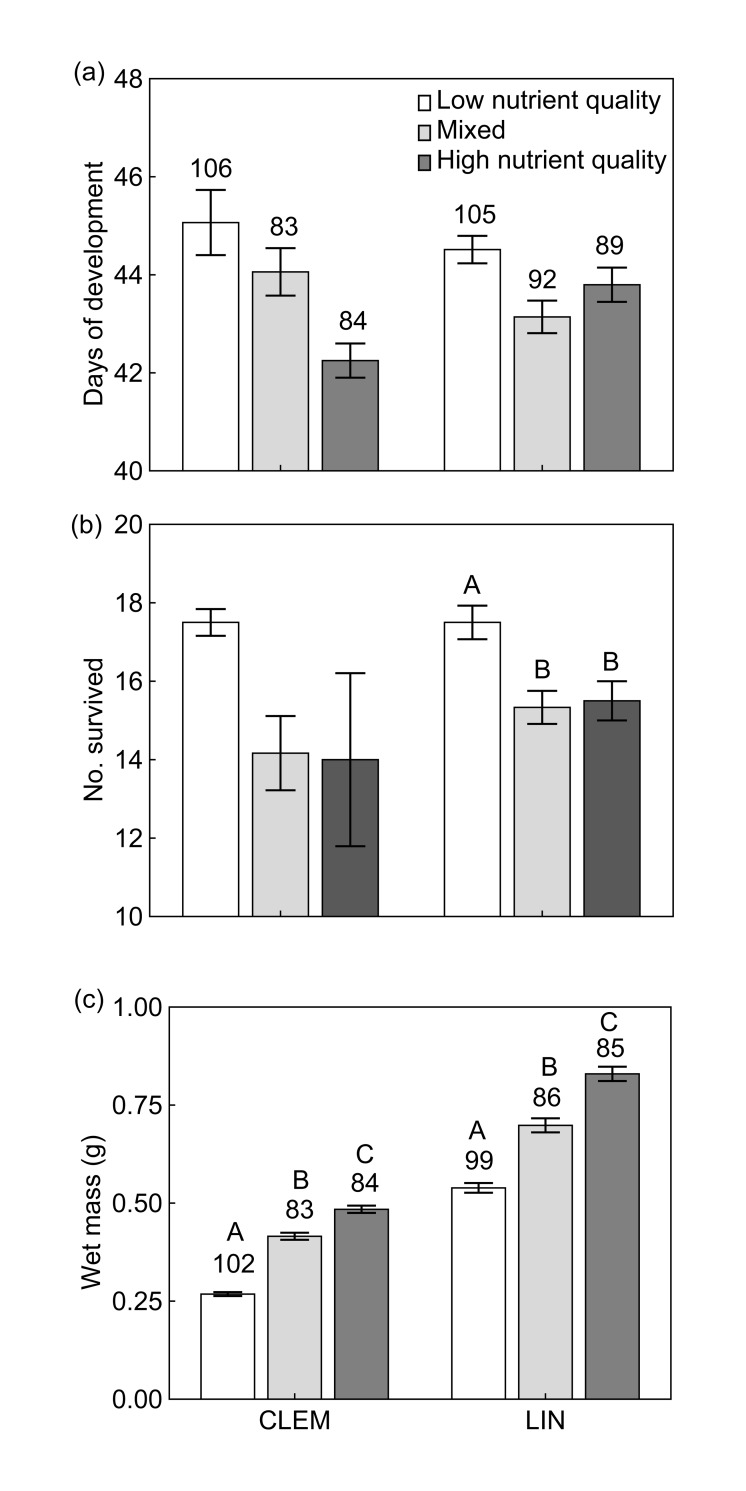
Anuran fitness. (a) Number of days of development, (b) number of surviving individuals, and (c) wet mass (g) for wood frogs at Clemson (CLEM) and Lindenwood (LIN) raised in the presence of low, high or mixed treatments. Sample sizes of individuals measured above each bar. Different upper case letters represent significant differences between treatments within a site. Error bars represents ±1 SD.

Anurans raised in low nutrient treatments had greater survival compared to high nutrient and mix treatments (Table 1; S1 Table), but this effect was driven by differences at Clemson (Fig 1B). There were no significant differences in developmental time across treatments (Table 1; Fig 1A). After accounting for differences in survival, at both Lindenwood and Clemson, anurans raised in high nutrient treatments were significantly larger than those raised in mixed and low nutrient treatments (Table 1; Fig 1C) and individuals raised in mixed treatments were significantly larger than those raised in low nutrient treatments (Table 1; Fig 1C). Individuals raised at Clemson were also significantly smaller than those raised at Lindenwood (Table 1; Fig 1C).

**Table 1:** Response to detritus quality.

Parameter		Survival					Days of development			Mass	
	df	MS	F	*P*	df	MS	F	*P*	MS	F	P
Survival	–	–	–	–	1	0.918	0.372	0.547	0.016	5.959	0.021
Site	1	7.111	1.096	0.304	1	0.013	0.457	0.504	0.760	288.522	≤ 0.001
Treatment	2	30.250	4.662	0.017	2	7.386	2.992	0.066	0.151	57.133	≤ 0.001
Site*Treatment	2	1.861	0.287	0.753	2	4.437	1.797	0.184	0.004	1.533	0.233
Error	30	6.489	–	–	29	2.468	–	–	0.003	–	–

General linear model results examining whether detritus nutrient quality (treatment) or site influence wood frog survival, and ANCOVAs examining whether mean number of days of development and mass at metamorphosis differed across sites, treatments, the interaction or a function of survival.

#### Biomass and nutrient export

**Fig 2:**
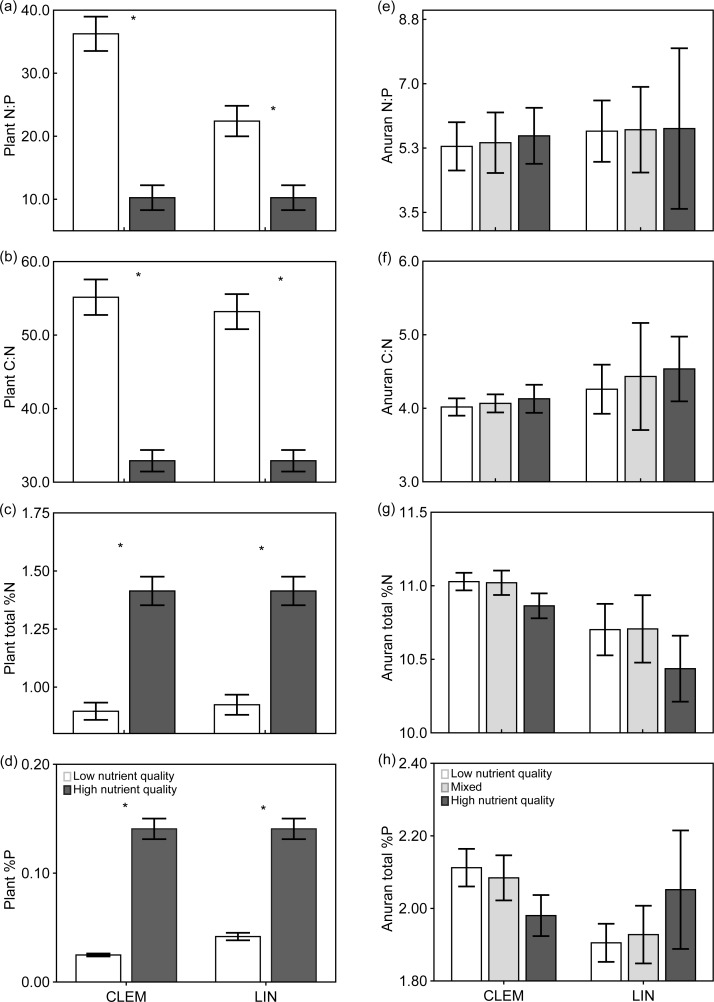
Litter and anuran stoichiometry. Mean (±1 SD) stoichiometry (%N, %P and C:N) of (a–d) low nutrient detritus (mixed native leaf litter), high nutrient detritus (purple loosestrife), and (e–h) metamorphic wood frogs raised in the presence of all treatments at Clemson (CLEM) and Lindenwood (LIN). * indicates significant differences between litter types plant stoichiometry. Amphibian %N and C:N was significantly different between sites. Error bars represent ±1 SD. Amphibian %N and C:N was significantly different across sites.

**Fig 3:**
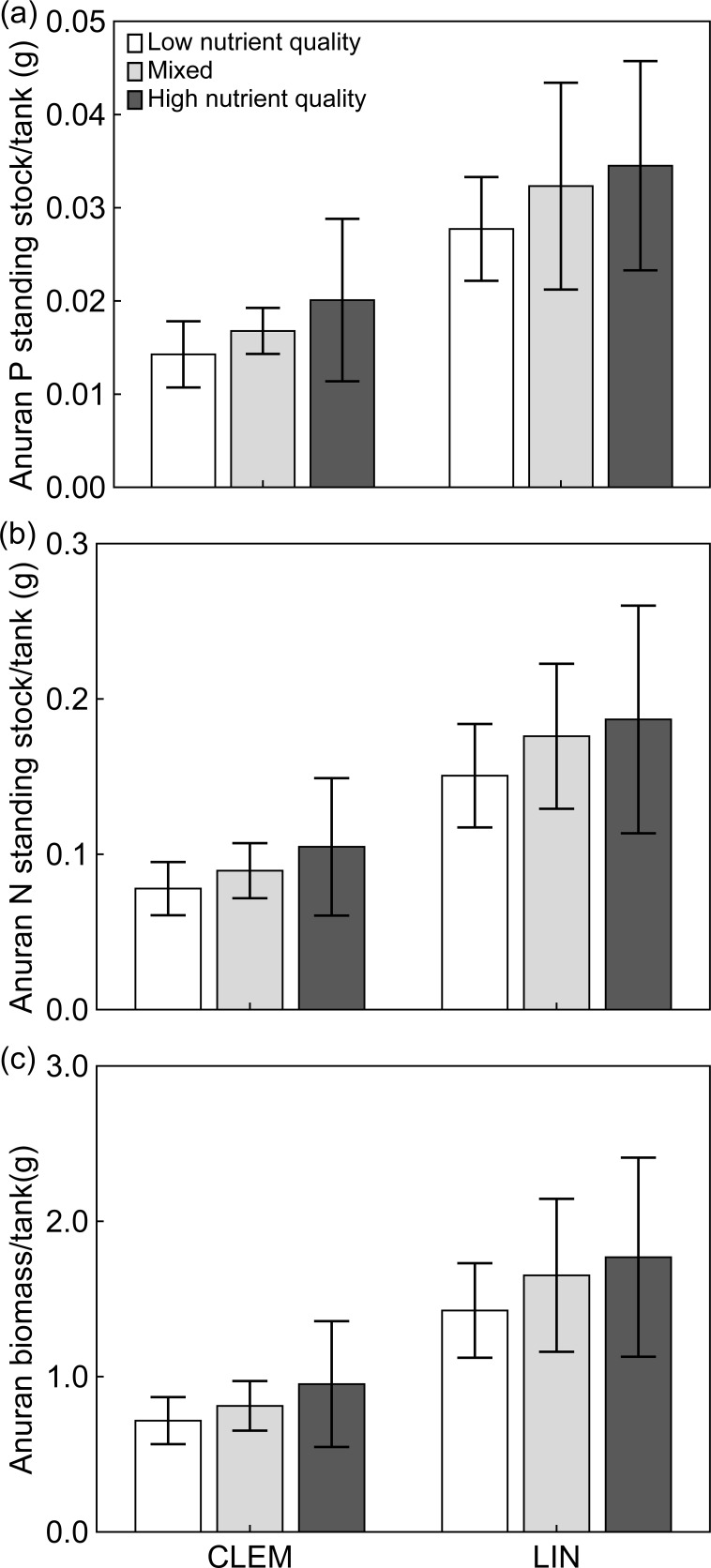
Standing stock of anurans. (a–b) Standing stock of N and P and (c) biomass of metamorphic wood frogs raised in the presence of all treatments at Clemson (CLEM) and Lindenwood (LIN). Error bars represent ±1 SD. Biomass and standing crop of N and P was significantly different across sites.

Nutrient content (N, P, C:N and N:P) of leaf litter differed across low and high nutrient treatments at Clemson (t-test; P: t = -12.119, *P≤*0.001; N: t = -7.217, *P≤*0.001; C:N: t = 7.891, *P≤*0.001) and Lindenwood (t-test; P: t = -9.822, *P≤*0.001; N: t = -6.518, *P≤*0.001; C:N: t = 7.263, *P≤*0.001; Fig 2A–2D; S1 Table) with high nutrient (purple loosestrife) litter having higher nutrient concentrations than low nutrient (native hardwood) litter. There were no differences between low nutrient litter between Clemson and Lindenwood (t-test; P: t = -1.008, *P* = 0.319; N: t = 0.556, *P* = 0.581; C:N: t = -0.096, *P* = 0.924). The stoichiometry (N, P, C:N and N:P) of metamorphic anurans at Clemson and Lindenwood was not significantly different across treatments (two-way ANOVA; *P* ≥ 0.05 for all nutrients; Fig 2E–2H), but two-way ANOVAs show larval anuran %N and C:N were significantly different across sites with %N being lower and C:N higher at Lindenwood (Table 2; Fig 2F and 2G). Non-parametric Mann-Whitney U tests show %N was significantly lower for low nutrient quality detritus (*P* = 0.050) at Lindenwood; however, there was no difference between high nutrient quality or mixed treatments (*P≥*0.05). Whole-body C:N was significantly higher at Lindenwood across treatments (*P≤*0.05). The biomass and standing stock of N and P in metamorphic anurans did not significantly differ across treatments, but did significantly differ across sites, with values from Lindenwood being 1.9, 1.8, and 1.9 times higher for biomass, N and P at Clemson (Table 3; Fig 3A–3C).

**Table 2:** Stoichiometry statistics.

Parameter		%N			%P			C:N			N:P		
	df	MS	F	*P*	MS	F	*P*	MS	F	P	MS	F	P
Site	1	4.561	7.546	0.007	0.341	1.874	0.173	4.107	27.343	≤0.001	3.732	2.629	0.107
Treatment	2	0.736	1.217	0.299	0.001	0.007	0.993	0.453	3.016	0.052	0.388	0.274	0.761
Site*Treatment	2	0.046	0.077	0.925	0.264	1.449	0.238	0.088	0.583	0.560	0.150	0.106	0.900
Error	30	0.600	–	–	0.182	–	–	0.150	–	–	1.419	–	–

Two-way ANOVA results examining the influence of site and treatment on wood frog whole-body %N, %P, C:N and N:P.

**Table 3:** Standing crop statistics.

Parameter		Biomass			Standing crop of N			Standing crop of P		
	df	MS	F	*P*	MS	F	*P*	MS	F	P
Site	1	5.600	35.070	≤0.001	0.058	31.121	≤0.001	0.002	30.174	≤0.001
Treatment	2	0.252	1.576	0.223	0.003	1.612	0.216	≤0.001	1.917	0.165
Site*Treatment	2	0.014	0.092	0.912	0.001	0.080	0.923	≤0.001	0.029	0.950
Error	30	0.160	–	–	0.002	–	–	0.002	–	–

Two-way ANOVA results examining the influence of site and treatment on wood frog biomass and standing crop of N and P.

## Discussion

### Effects of litter nutrient quality on anuran development, survival and size

The drivers of differences in anuran fitness proxies in our study appear to, in part, be a result of variation in nutrient quality and reduced competition. Several studies have found correlations between higher detritus nutrient quality (e.g., low C:N) and anuran development [4,6,23,24], regardless of if the detritus was derived from a native or invasive plant. Purple loosestrife used in our study had significantly higher nutrient content and lower C:N and N:P (Fig 2). Although these large differences did not translate to measurable increased nutrient content in larval anurans (Fig 2), wood frogs in purple loosestrife treatments that contained higher nutrient content were significantly larger at metamorphosis.

Our data suggest larval anurans grew larger and faster when raised with high nutrient quality (either as the sole resource or mixed with low nutrient quality litter) and that anurans raised with high nutrient quality litter (purple loosestrife; mixed or high treatments) did have reduced survival at both sites. One consistency across sites that could explain the reduction in survival is secondary compounds contained in purple loosestrife (our high nutrient quality detritus). Although not measured in this study, negative effects of compounds from invasive plants on amphibian larvae have been documented [43,44]. The effects from purple loosestrife chemicals in particular on anurans was the focus of one such study by Maerz et al. [2] where the effects of purple loosestrife litter extracts was hypothesised to decrease survival as a consequence of a disruption of oxygen transfer due to phenolics binding to gill cells. The decrease in survival of larval wood frogs when raised with purple loosestrife likely reduced competition among surviving congeners. This reduction in competition likely increased the ability to forage on high nutrient quality purple loosestrife, or associated algae, for those individuals that could tolerate the phenolics. Wood frog tadpoles are known to consume a variety of resources; including plant material, algae and zooplankton [22,33,34]; therefore, we cannot speculate on consumption, and potential contribution to growth and survival, of these resources within treatments across our study sites (algal and zooplankton biomass were not measured). Since body mass was higher in high nutrient quality and mixed treatments after accounting for survival, we believe the positive influence of nutrient quality of purple loosestrife has measurable impacts on the size of wood frog tadpoles.

### Effect of reduced survival and increased size on anuran biomass and standing stock

Biomass export of anurans is driven by a combination of survival and growth, and detritus nutrient quality affects the influence of both of these drivers [3,23,25,29]. Do the factors that negatively influence metamorphic anuran export (i.e., secondary plant compounds), and those that positively influence it (i.e., high nutrient quality), offset one another? For example, a particular plant with high phenolics and high nutrient quality could reduce survival of anuran larvae, but produce larger larvae in return–thus creating a net zero or net gain in anuran biomass. Several studies have found direct relationships between higher leaf and grass detritus quality and increased anuran biomass export [30,31]. Most recently, Earl et al. [32] showed high nutrient content and high secondary compounds of white oak (*Quercus alba*) resulted in measurable and distinct increases in larval anuran size, but decreases in survival. The resulting biomass export was not appreciably different from less toxic plant species in the study. In our study, anuran biomass and standing stock of N and P were not significantly different in treatments that contained high nutrient quality litter compared to low nutrient quality litter (Fig 3). However, we did see differences in biomass and standing crop of limiting nutrients across sites, which is likely due to the variation in size of Clemson and Lindenwood anurans (Fig 1). In wood frogs, phenotypic variation of size in post-metamorphic individuals exists at local scales and growth of larval wood frogs does differ across a latitudinal gradient (greater in northern latitudes) and temperature regimes [45], and it appears these variations can lead to differences in export biomass and standing crop of nutrients. Variations in biomass export of anurans has been shown to be influenced by canopy cover and plant litter input [31,32], but our study shows export may be influenced by phenotypic variation of post-metamorphic anuran size.

At the ecosystem level, the decrease in survival and increase in size of larval anurans raised with purple loosestrife could have several consequences. Within lentic systems, larval anurans regulate primary productivity, alter periphyton and algal community structure, serve as prey for higher trophic levels and as sinks of organic N [46,47]. Furthermore, decreased survival in larval anurans reduces the density of post-metamorphic individuals entering the adjacent landscape. Success of post-metamorphic individuals to the point of reproduction is imperative to maintain positive population growth, and the degree of post-metamorphic success could influence metacommunity dynamics across a region [48]. Post-metamorphic survival of anurans can be dictated by size prior to metamorphosis. For example, several studies have shown post-metamorphic survival significantly increases with increased size of post-metamorphic individuals and decreased developmental time–in our study size was greater in individuals raised in treatments with high nutrient quality litter and developmental time was marginally significant. Additionally, export of post-metamorphic anurans can be considered a flux of energy and nutrients into surrounding terrestrial habitat [49,50]. When considering anuran-driven ecosystem functions within lentic and adjacent systems, a decrease in survival will limit the number of individuals present; however, if the individuals are larger due to higher nutrient quality of plant material (invasive or non-invasive) some ecological functions could remain unchanged across wetlands with high nutrient quality plants.

Future investigations would benefit from combining our multi-regional approach with other, more realistic experimental venues. Such an approach would allow researchers to evaluate animal responses within more complex natural environments [51,52] where issues of plant competition, *in situ* litter quality, and animal foraging behavior may differ from mesocosm trials. Additionally, studies of this topic may benefit from addressing the influence of wetland environments on the terrestrial life stages of anurans (i.e. carryover or legacy effects). Finally, we know very little about the interaction between variation in plant quality and other stressors both local (e.g., predation and competition) and global (e.g., climate change and infectious diseases). Sorting out such interactions will be critical since stressors are not isolated from one another in natural ecosystems.

## Supporting Information

S1 Table Raw fitness and stoichiometric data. Raw values of number of metamorphing individuals, mean number of days in each tank, mean body mass (mg), total %N and %P, mean biomass per tank (g) and mean standing crop of N and P (g) for tadpoles across all treatments at Clemson University and Lindenwood University. Values of total %P, %N, C:N and N:P of homogonized native hardwood litter and purple loosestrife from Clemson University, Lindenwood University and Indiana.
(XLSX)
